# Household Income Differences and Variations in Management and Outcomes of Patients With Acute Pulmonary Embolism

**DOI:** 10.1016/j.jscai.2025.103654

**Published:** 2025-09-30

**Authors:** Mridul Bansal, Aryan Mehta, Emil D. Missov, Vinayak Nagaraja, J. Dawn Abbott, Saraschandra Vallabhajosyula

**Affiliations:** a Department of Medicine, East Carolina University Brody School of Medicine, Greenville, North Carolina; b Department of Medicine, University of Connecticut School of Medicine, Farmington, Connecticut; c Brown University Health Cardiovascular Institute, Providence, Rhode Island; d Division of Cardiology, East Carolina Heart Institute, Greenville, North Carolina; e Department of Cardiovascular Medicine, Mayo Clinic, Rochester, Minnesota; f Division of Cardiology, Department of Medicine, Warren Alpert Medical School of Brown University, Providence, Rhode Island

**Keywords:** critical care cardiology, health care outcomes, household income, pulmonary embolism, socioeconomic disparities

There are numerous studies in acute cardiovascular conditions showing variations in care that cannot be solely explained by clinical factors.^1^ Individuals from the lower socioeconomic status (SES) tend to belong more to minority races, have higher burden of chronic illness, greater exposure to environmental hazards, and have less or minimal access to health care.^1^ There are relatively sparse data on the variation of pulmonary embolism (PE) outcomes based on SES of the patient, which forms an essential component of the social determinant of health. In this context, we aimed to use a national database to assess variations in health care delivery and outcomes in patients with PE, specifically based on their SES.

The Healthcare Cost and Utilization Project National Inpatient Sample (HCUP-NIS) is an all-payer database of inpatient hospital stays, which encompasses a 20% stratified sample of these admissions in the United States. The database is deidentified, and therefore, institutional review board approval was not sought.^2^ Using the HCUP-NIS data from January 1, 2016, to December 31, 2021, we identified all adult (≥18 years) urgent or emergent admissions with a primary diagnosis of PE (International Classification of Diseases 10.0 Clinical Modification: I260, I2601, I2602, I2609, I2690, I2692, I2693, I2694, and I2699) and stratified into 4 quartiles of medical household income representing SES of the admissions.^3^ HCUP-NIS classifies the median household income for patient’s ZIP Code into 4 quartiles: 0th to 25th percentile, 26th to 50th percentile, 51st to 75th percentile, and 76th to 100th percentile. These values are derived from ZIP Code-demographic data obtained from Claritas. They are updated annually, and thus, the ranges of each quartile vary year by year. Detailed description of this income distribution is available via the HCUP-NIS.^2^ Acute noncardiac organ failure, in-hospital complications, and PE interventions codes are described in Supplemental Table S1. The primary outcome was in-hospital mortality, and secondary outcomes included rates in PE interventions, total hospitalization costs, discharge dispositions, and length of hospital stay in admissions belonging to different quartiles. All analysis were done using Stata 16.0 (StataCorp LLC).

**Figure 1:**
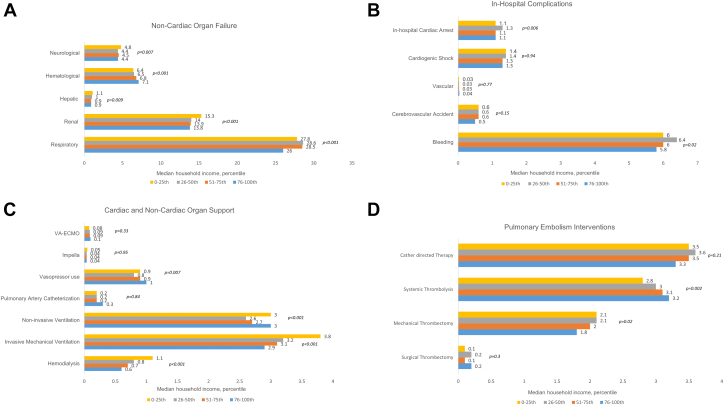
**Stratification by socioeconomic status.** (A) Acute organ failure and complications of pulmonary embolism admissions stratified by socioeconomic status. (B) In-hospital complications of pulmonary embolism admissions stratified by socioeconomic status. (C) Cardiac and noncardiac organ support of pulmonary embolism admissions stratified by socioeconomic status. (D) Interventions of pulmonary embolism admissions stratified by socioeconomic status.

During January 1, 2016, to December 31, 2021, 1,078,109 PE admissions were identified across the United States. Among these, 28.4% (307,114) belonged to the 0th to 25th quartile of median household income, 26.4% (284,939) to the 26th to 50th quartile, 24.6% (266,134) to the 51th to 75th quartile, and 20.4% (219,919) to the 76th to 100th quartile. Compared with the highest quartile (76th to 100th), the admissions in the other income quartiles were on average younger, were females, were of non-White race, had higher comorbidity, had Medicaid as primary payer, were from the Southern region, and received care at larger hospitals (all *P* < .001) (Supplemental Table S2). Compared with the highest quartile (76th to 100th), median household income, the rates of noncardiac organ failure, and use of noncardiac organ support were higher in other groups; however, in-hospital complications such as cardiac arrest, cardiogenic shock, bleeding, and vascular complications were largely comparable (Figure 1A-C). Admissions in the groups with lower median household income quartile had lower rates of systemic thrombolysis but had comparable rates of other PE interventions like surgical thrombectomy, mechanical thrombectomy, and catheter-directed therapy when contrasted with those in the highest median household income quartile (Figure 1D). Unadjusted in-hospital mortality for PE admissions was higher in lower income quartiles (0th to 25th, 3.3%; 26th to 50th, 3.1%; 51st to 75th, 3.0%; and 76th to 100th, 3.0%; *P* = .01), but these differences did not persist after adjustment using relevant clinical and demographic variables (0th to 25th, referent category; 26th to 50th: odds ratio [OR], 0.97; 95% CI, 0.88-1.07; *P* = .66; 51st to 75th: OR, 0.96; 95% CI, 0.86-1.06; *P* = .45; 76th to 100th: OR, 0.95; 95% CI, 0.85-1.07; *P* = .47). Compared with highest median household income quartile (76th to 100th), admissions belonging to lower median household income quartiles had longer length of stay and less frequent discharges to home (all *P* < .001) (Supplemental Tables S3 and S4).

In this nationwide study, we noted that there existed significant differences in the demographic and clinical characteristics between the SES categories. The admissions from the lowest income quartile had higher burden of comorbidity, higher rates of organ failure, and comparable rates of PE intervention. Previous studies have reported how non-White races, particularly Black population, have higher percentage of PE hospitalization rates, especially from the lower SES.^4^ Similarly, Farmakis et al^4^ noted that the lowest income earners made the highest proportion of PE admissions (∼29%). A recent study reported that compared with the highest household income quartile, admissions from the lowest income quartile were less likely to receive these therapies in high-risk PE (OR, 0.86; 95% CI, 0.75-0.99).^4^ Furthermore, another analysis reported that the SES-disadvantaged population are less likely to receive advance therapies for PE compared with nondisadvantaged population (11% vs 12.1%).^5^ Notably, there is heterogeneity in in-hospital mortality rates that makes it difficult to establish a causal link between management decisions and PE outcomes, even though lower utilization of PE therapies in lower SES patients may be correlated. Some reports have noted differences in mortality rates based on SES, while others have not.^4^^,^^5^ In this study, patients admitted from the lowest income quartile were sicker but had lower rate of systemic thrombolysis, inferring toward variations in utilization of PE interventions. However, given that all difference in care does not necessarily translate to disparities and the variation in rates of PE therapies may be justified based on the state of the science for them, more extensive research needs to be undertaken to help quantify the actual burden of these SES disparities. Finally, it is important to note that the HCUP-NIS data set is only available until 2021, which predates the widespread use of catheter-directed therapies for acute PE.

In summary, despite comparable in-hospital mortality, acute PE admissions with a lower SES had higher rates of noncardiac organ failure and greater resource utilization. Further studies are needed to better delineate the rationale for these disparities in outcomes and help aid in the standardization to improve health care delivery in the socially disadvantaged patient population.
