# Supplementary material for: Household Income Differences and Variations in Management and Outcomes of Patients With Acute Pulmonary Embolism
Source: J Soc Cardiovasc Angiogr Interv. 2025 Sep 30;5(3 Suppl):103654. doi: 10.1016/j.jscai.2025.103654 (PMC13112804; doi:10.1016/j.jscai.2025.103654)
Supplement: Supplementary Tables S1-S4 [file mmc1.docx]

**Influence of Household Income Disparities in Management and Outcomes of Patients Admitted with Pulmonary Embolism**

Mridul Bansal, MD; Aryan Mehta, MD; Emil D Missov, MD; Vinayak Nagaraja, MD; J Dawn Abbott, MD; Saraschandra Vallabhajosyula, MD MSc

**Supplementary Table S1. Administrative codes used to identify diagnoses and procedures**

| **Diagnosis/Procedure** | **Administrative Code** |
| --- | --- |
| Pulmonary Embolism | I2601, I2602, I2609, I2690, I2692, I2693, I2694, I2699 |
| Respiratory failure | J96.0, J96.00, J96.01, J96.02, J96.20, J96.21, J96.22, J96.9, J969.0, J96.91, J96.92, J80.0, R06.00, R06.03, 5A1935Z, 5A1945Z, 5A1955Z, R09.20 |
| Renal failure | N17.0, N17.1, N17.2, N17.8, N17.9 |
| Hepatic failure | K72.00, K76.2, K72.90, K72.01, K72.91, K71.6, K75.2, K75.9, K76.3 |
| Hematologic failure | D68.32, D68.4, D65.0, D68.8, D68.9, D69.6, D69.59 |
| Bleeding complications | I85.0, K25.0, K25.2, K25.4, K25.6, K27.0, K27.2, K27.4, K27.6, K28.0, K28.2, K28.48, K29.0, K62.5, K92.0 K92.1, K92.2, D62.0, H11.3, H35.6, H43.1, H45.0, H92.2, J94.2, K66.1, M25.0, N02.0, N92.0, N92.1, N92.4, N93.8, N93.9, N95.0, R04.0, R04.1, R04.2, R04.8, R04.9, R31.0, R58.0 |
| Neurologic failure | R40.0, R40.1, R40.2211, R40.2212, R40.2213, R40.2214, R40.222, R40.2220, R40.2221, R40.2222, R40.2223, R40.2224, R40.223, R40.2230, R40.2231, R40.2232, R40.2233, R40.2234, R40.224, R40.2240, R40.2241, R40.2242, R40.2243, R40.2244, R40.225, R40.2250, R40.2251, R40.2252, R40.2253, R40.2254, R40.231, R40.2310, R40.2311, R40.2312, R40.2313, R40.2314, R40.232, R40.2320, R40.2321, R40.2322, R40.2323, R40.2324, R40.233, R40.2330, R40.2331, R40.2332, R40.2333, R40.2334, R40.234, R40.2340, R40.2341, R40.2342, R40.2343, R40.2344, R40.235, R40.2350, R40.2351, R40.2352, R40.2353, R40.2354, R40.236, R40.2360, R40.2361, R40.2362, R40.2363, R40.2364, R40.241, R40.2410, R40.2411, R40.2412, R40.2413, R40.2414, R40.242, R40.2420, R40.2421, R40.2422, R40.2423, R40.2424, R40.243, R40.2430, R40.2431, R40.2432, R40.2433, R40.2434, R40.244, R40.2440, R40.2441, R40.2442, R40.2443, R40.2444, R40.2, R40.20, R40.21, R40.22, R40.23, R40.24, R40.3, R40.4, R40.211, R40.2110, R40.2111, R40.2112, R40.2113, R40.2114, R40.212, R40.2120, R40.2121, R40.2122, R40.2123, R40.2124, R40.213, R40.2130, R40.2131, R40.2132, R40.2133, R40.2134, R40.214, R40.2140, R40.2141, R40.2142, R40.2143, R40.2144, R40.221, R40.2210, G93.40, G93.41, G93.49, I67.83, F05, F06.2, F06.0, F06.30, F06.4, F06.1, F53, F06.8, G93.1, N17.0, N17.1, N17.2, N17.8, N17.9 |
| Vascular complications requiring surgery | 04QC, 04QC0, 04QC0Z, 04QC0ZZ, 04QC3, 04QC3Z, 04QC3ZZ, 04QC4, 04QC4Z, 04QC4ZZ, 04QD, 04QD0, 04QD0Z, 04QD0ZZ, 04QD3, 04QD3Z, 04QD3ZZ, 04QD4, 04QD4Z, 04QD4ZZ, 04QE, 04QE0, 04QE0Z, 04QE0ZZ, 04QE3, 04QE3Z, 04QE3ZZ, 04QE4, 04QE4Z, 04QE4ZZ, 04QF, 04QF0, 04QF0Z, 04QF0ZZ, 04QF3, 04QF3Z, 04QF3ZZ, 04QF4, 04QF4Z, 04QF4ZZ, 04QH, 04QH0, 04QH0Z, 04QH0ZZ, 04QH3, 04QH3Z, 04QH3ZZ, 04QH4, 04QH4Z, 04QH4ZZ, 04QJ, 04QJ0, 04QJ0Z, 04QJ0ZZ, 04QJ3, 04QJ3Z, 04QJ3ZZ, 04QJ4, 04QJ4Z, 04QJ4ZZ, 04QK, 04QK0, 04QK0Z, 04QK0ZZ, 04QK3, 04QK3Z, 04QK3ZZ, 04QK4, 04QK4Z, 04QK4ZZ, 04QL, 04QL0, 04QL0Z, 04QL0ZZ, 04QL3, 04QL3Z, 04QL3ZZ, 04QL4, 04QL4Z, 04QL4ZZ |
| Non-cardiac organ support | 5A19054, 5A1935Z, 5A1945Z, 5A1955Z, 5A09357, 5A09457, 5A09557, 5A09358, 5A09458, 5A09558,5A0935Z, 5A0945Z, 5A0955Z, 3E030XZ, 3E033XZ, 3E040XZ, 3E043XZ |
| Systemic thrombolysis | 3E03317, 3E04317 |
| Catheter directed therapy | 3E06317, 6A750Z5 ,6A750Z6, 6A750Z7, 6A750ZZ, 6A751Z5,6A751Z6, 6A751Z7, 6A751ZZ |
| Mechanical thrombectomy | 02CP3ZZ, 02CQ3ZZ, 02CR3ZZ |
| Surgical thrombectomy | 02CP0ZZ, 02CQ0ZZ, 02CR0ZZ |

**Supplementary Table S2: Baseline and hospital characteristics of pulmonary embolism admissions stratified by socioeconomic status**

| **Baseline Characteristics** | | | **Median household income, percentile** | | | | ***P* value** |
| --- | --- | --- | --- | --- | --- | --- | --- |
|  |  |  | **0-25^th^**  **(N=307,114)** | **26-50^th^**  **(N=284,939)** | **51-75^th^**  **(N=266,134)** | **76-100^th^ (N=219,919)** |  |
| **Proportion** | | | 28.4 | 26.4 | 24.6 | 20.4 | - |
| **Age (in years)** | | | 61.4 ± 0.07 | 63.2 ± 0.07 | 63.6 ± 0.07 | 64.5 ± 0.08 | 0.01 |
| **Female** | | | 52.5 | 51.7 | 51.6 | 50.9 | <0.001 |
| **Race** | | White | 55.0 | 72.5 | 74.5 | 77.2 | <0.001 |
|  |  | Black | 32.6 | 15.8 | 13.6 | 10.7 | <0.001 |
|  |  | Hispanic | 7.2 | 5.6 | 5.3 | 4.2 | <0.001 |
|  |  | Asian | 0.4 | 0.6 | 1.1 | 2.1 | <0.001 |
|  |  | Native American | 0.5 | 0.3 | 0.2 | 0.2 | <0.001 |
|  |  | Others | 1.8 | 1.7 | 2.0 | 2.7 | <0.001 |
|  |  | Missing | 2.4 | 3.2 | 2.9 | 2.5 | <0.001 |
| **Weekend admission** | | | 24.2 | 23.8 | 23.4 | 23.1 | <0.001 |
| **Elixhauser comorbidity index** | | | 4.5 ± 0.01 | 4.4 ± 0.01 | 4.3 ± 0.01 | 4.2 ± 0.01 | <0.001 |
| **Primary payer** | Medicare | | 50.8 | 53.4 | 52.0 | 52.2 | <0.001 |
|  | Medicaid | | 17.9 | 12.2 | 6.6 | 6.5 |  |
|  | Private | | 22.4 | 27.0 | 31.1 | 36.6 |  |
|  | Self-pay | | 5.5 | 4.1 | 3.3 | 2.3 |  |
|  | No Charge | | 0.4 | 0.2 | 0.2 | 0.1 |  |
|  | Others | | 2.8 | 2.8 | 2.6 | 2.1 |  |
| **Hospital region** | Northeast | | 12.3 | 15.4 | 20.8 | 28.8 | <0.001 |
|  | Midwest | | 24.5 | 28.6 | 26.6 | 19.7 |  |
|  | South | | 51.3 | 39.6 | 32.4 | 27.3 |  |
|  | West | | 11.6 | 16.3 | 20.8 | 24.0 |  |
| **Hospital size** | Small | | 18.0 | 22.2 | 23.3 | 24.0 | <0.001 |
|  | Medium | | 27.5 | 28.7 | 29.4 | 32.5 |  |
|  | Large | | 54.3 | 48.9 | 47.1 | 43.3 |  |
| **Hospital location/ teaching status** | Rural | | 15.4 | 12.2 | 4.4 | 0.8 | <0.001 |
|  | Urban non-teaching | | 16.7 | 22.6 | 23.9 | 22.9 |  |
|  | Urban teaching | | 67.8 | 65.1 | 71.8 | 76.2 |  |

**Represented as:** Number (percentage) or mean ± standard deviation

**Supplementary Table S3: Clinical outcomes of pulmonary embolism admissions stratified by socioeconomic status**

|  | **Median household income, percentile** | | | | ***P* value** |
| --- | --- | --- | --- | --- | --- |
|  | **0-25^th^**  **(N=307,114)** | **26-50^th^**  **(N=284,939)** | **51-75^th^**  **(N=266,134)** | **76-100^th^ (N=219,919)** |  |
| In-hospital mortality (%) | 3.3 | 3.1 | 3.0 | 3.0 | 0.01 |
| Length of stay (in days) | 4.6 ± 0.02 | 4.3 ± 0.02 | 4.2 ± 0.2 | 4.1 ± 0.2 | <0.001 |
| Total Charge (in $) | 52,214 ± 445 | 50,302 ± 422 | 51,106 ± 419 | 55,341 ± 648 | <0.001 |
| **Discharge Disposition** |  | | | | |
| Routine | 64.0 | 64.8 | 65.0 | 64.2 | <0.001 |
| Skilled nursing facility | 13.1 | 13.5 | 13.5 | 13.5 |  |
| Home health care | 15.9 | 15.2 | 15.7 | 16.8 |  |
| Against medical advice | 1.2 | 0.8 | 0.7 | 0.5 |  |

**Represented as:** Number (percentage) or mean ± standard deviation

**Supplementary Table S4: Multivariable regression for in-hospital mortality in pulmonary embolism**

| **Variable** | **Odds Ratio** | **P** | **95% Confidence Interval** | |
| --- | --- | --- | --- | --- |
|  |  |  | **Lower Limit** | **Upper Limit** |
| **Median household income percentile** | *0-25th percentile reference category* | | | |
| 26-50^th^ | 0.97 | 0.66 | 0.88 | 1.07 |
| 51-75^th^ | 0.96 | 0.45 | 0.86 | 1.06 |
| 76-100^th^ | 0.95 | 0.47 | 0.85 | 1.07 |
| **Female** | 1.06 | 0.10 | 0.98 | 1.14 |
| **Age** | 1.02 | <0.001 | 1.02 | 1.03 |
| **Weekend admission** | 0.94 | 0.14 | 0.86 | 1.02 |
| **Race** |  | | | |
| White | 0.86 | 0.13 | 0.72 | 1.04 |
| Black | 0.76 | 0.01 | 0.62 | 0.93 |
| Asian | 1.46 | 0.26 | 1.04 | 2.03 |
| Hispanic | 0.91 | 0.43 | 0.71 | 1.15 |
| Native | 1.17 | 0.55 | 0.68 | 2.03 |
| Elixhauser comorbidity index | 1.09 | <0.001 | 1.07 | 1.11 |
| **Hospital location and teaching status** | *Rural reference category* | | | |
| Urban Non-teaching | 0.81 | 0.008 | 0.69 | 0.94 |
| Urban teaching | 1.01 | 0.88 | 0.88 | 1.15 |
| **Hospital region** | *Northeast reference category* | | | |
| Midwest | 0.73 | <0.001 | 0.65 | 0.82 |
| South | 0.86 | 0.007 | 0.78 | 0.96 |
| West | 0.83 | 0.002 | 0.73 | 0.93 |
| **Hospital Bed Size** | *Small reference category* | | | |
| Medium | 1.07 | 0.2 | 0.96 | 1.19 |
| Large | 1.26 | <0.001 | 1.14 | 1.40 |
| **Primary expected payer** | *Medicare reference category* | | | |
| Medicaid | 1.13 | 0.10 | 0.97 | 1.31 |
| Private | 1.04 | 0.06 | 0.99 | 1.22 |
| Self-pay | 1.49 | <0.001 | 1.21 | 1.84 |
| No Charge | 1.01 | 0.977 | 0.4 | 2.43 |
| Others | 1.24 | 0.07 | 0.98 | 1.58 |
| **Respiratory failure** | 3.02 | <0.001 | 2.78 | 3.28 |
| **Renal failure** | 1.66 | <0.001 | 1.52 | 1.81 |
| **Hepatic failure** | 1.12 | 0.28 | 0.90 | 1.38 |
| **Neurologic failure** | 1.96 | <0.001 | 1.76 | 2.19 |
| **Bleeding Complication** | 0.71 | <0.001 | 0.61 | 0.83 |
| **Hemodialysis** | 0.78 | 0.14 | 0.57 | 1.08 |
| **Vasopressors use** | 1.58 | <0.001 | 1.28 | 1.95 |
| **Outside hospital cardiac arrest** | 1.07 | 0.88 | 0.39 | 2.88 |
| **In-hospital cardiac arrest** | 15.9 | <0.001 | 12.08 | 19.8 |
| **Cardiogenic shock** | 1.69 | <0.001 | 1.41 | 2.03 |
| **Vascular complications** | 0.05 | <0.001 | 0.01 | 0.17 |
| **Non-invasive ventilation** | 2.15 | <0.001 | 1.88 | 2.47 |
| **Invasive mechanical ventilation** | 13.08 | <0.001 | 11.68 | 14.64 |
| **Cather directed therapy** | 0.55 | <0.001 | 0.45 | 0.68 |
| **Systemic thrombolysis** | 1.07 | 0.39 | 0.91 | 1.25 |
| **Mechanical thrombectomy** | 1.09 | 0.45 | 0.85 | 1.4 |
| **Surgical thrombectomy** | 0.45 | 0.39 | 0.47 | 1.34 |
| **Pulmonary artery catheterization** | 0.86 | 0.62 | 0.47 | 1.57 |
| **VA-ECMO** | 1.15 | 0.64 | 0.62 | 2.14 |
| **Impella** | 2.38 | 0.03 | 1.07 | 5.2 |
